# Powerless or Powerful? Healthcare Professionals' Construction of Agency in Patient and Family Engagement Accounts

**DOI:** 10.1111/hex.70743

**Published:** 2026-07-01

**Authors:** Polina Mesinioti, Laura Sheard, Sarah Hampton, Gemma Louch, Carl Macrae, Jane O'Hara

**Affiliations:** ^1^ Department of Health Sciences University of York York UK; ^2^ School of Health Sciences University of Manchester Manchester UK; ^3^ University College London (UCL) London UK; ^4^ School of Healthcare University of Leeds Leeds UK; ^5^ Nottingham University Business School University of Nottingham Nottingham UK; ^6^ The Healthcare Improvement Studies (THIS) Institute University of Cambridge Cambridge UK

**Keywords:** agency, discourse analysis, health policy, patient and family engagement, patient safety, patient safety incident

## Abstract

**Background:**

Patient safety incidents cause substantial harm globally, with affected patients and families often experiencing additional ‘compounded harm’ from inadequate organisational responses. Despite policy imperatives emphasising engagement as essential for safety improvement, significant gaps persist in the National Health Service (NHS), where involvement in Serious Incident (SI) investigations is often overlooked or treated as a p2assive process. While previous qualitative research has primarily used thematic analysis, discourse analytic approaches can offer deeper insights into nuanced patterns of meaning.

**Design/Objective:**

We conducted discourse analysis on 49 semi‐structured interviews with healthcare professionals across six NHS Trusts to examine how staff construct agency in their accounts of engaging patients and families in SI investigations and how these discourses are linked to the broader organisational context.

**Results:**

Findings illustrate two prevailing but contested discourses: one depicting staff engagement with patients and families as inconsistent and limited, and the other emphasising their integral role in the investigation process. Interviewees framed themselves as either powerless or powerful, aligning with these respective discourses, while organisational factors, such as professional roles and work relationships, also influenced those constructions.

**Discussion:**

A focus on the linguistic construction of agency, control and responsibility provides a powerful lens for understanding persistent gaps in patient and family engagement and highlights the value of incorporating discourse analytic approaches into health policy development and implementation.

**Patient or Public Contribution:**

A Citizens' Panel (*n* = 16) was involved throughout the broader research programme, ensuring public accountability; supporting wider discussions about the emergent findings of the research and their implications for fairness, equality, diversity and inclusion; and supporting the dissemination of findings in creative and accessible ways.

## 1 Introduction

Patient safety is a persistent healthcare issue around the world, with one in every 10 patients being harmed in healthcare, and more than 3 million deaths occuring annually due to unsafe care [1]. In England, more than 2 million patient safety events are recorded annually [2]. Patient safety incidents can have devastating emotional and physical consequences on patients, families and carers, who often suffer additional, ‘compounded harm’ [3] because of a lack of openness and support following patient safety incidents and poor‐quality investigations. Patient involvement is a valuable source of information, not only complementing existing patient safety measurements [4] but also promoting an open culture and helping ensure the safety of future care [5].

Engagement with patients, carers and families is often overlooked in Serious Incident (SI) investigations (for the definition of SIs, see Section 1.1). In England, despite the ‘Being Open’ framework [6] providing guidance on communication with patients and families, significant gaps persist. A recent analysis of 43 NHS SI investigation policies [7] found that support or involvement of those affected was either absent or described as passive.

### 1.1 The Serious Incident Framework

In England, the Serious Incident Framework (SIF) [8] served as the first national policy governing patient safety incidents across all NHS Trusts in the United Kingdom until 2022, when it was replaced by the Patient Safety Incident Response Framework (PSIRF) [9]. The SIF outlined expectations for the reporting and investigation of SIs, with SIs being defined as ‘events in health care where the potential for learning is so great, or the consequences to patients, families and carers, staff or organisations are so significant, that they warrant using additional resources to mount a comprehensive response’ [8, p. 12].

Directly relevant to our work are the policy's explicit references to engaging and supporting those affected by incidents, which emphasise the principles of *honesty*, *openness* and *transparency*. The policy requires that patients, families and carers be promptly informed about investigations, given opportunities to contribute to the process and terms of reference, have access to findings, and be kept updated about any delays with clear explanations. Despite the policy's explicit focus on meaningfully involving patients and families throughout the investigation process, existing metrics and research on the SIF reveal a disconnect between the policy's intentions and the organisational realities, as detailed below.

### 1.2 Research on Patient and Family Engagement and Current Gaps

Current evidence on the SIF's effectiveness in engaging patients and families stems from reviews of incident data, complaints and investigation reports. An examination of 74 Acute Trusts revealed limited patient/family involvement in investigations, few opportunities to discuss reports, and a lack of evidence that patients/families received report copies despite policy requirements [10]. Similarly, HCI Care [11] reported poor communication, lack of open disclosure, and unstructured approaches—findings that demonstrate the inconsistent approaches Trusts take in engaging patients, families and carers.

While reviews of incident data provide useful insights, they offer limited perspectives on the lived experiences of healthcare professionals managing patient safety incidents. Qualitative research has begun to address this gap, but, despite widespread recognition of the importance of patient and family involvement, significant gaps persist. Ramsey et al.'s (2022) review of qualitative evidence on patient and family involvement identified insufficient multidisciplinary input, high workloads, staff turnover, inadequate training and litigation concerns. Their more recent interview study [12] found that patients and families lacked power, knowledge and support to navigate investigations, resulting in compounded harm.

Although it is now well known that involvement is inconsistent and often inadequate, we lack understanding of *how* healthcare professionals make sense of their role in this process—particularly how they construct their own agency and responsibility when engaging (or failing to engage) patients and families. Most qualitative studies have employed thematic/content analysis, identifying *what* barriers exist but not *how* professionals discursively position themselves in relation to these challenges; we address this gap here.

### 1.3 Research Aim

The aim of this study was to examine how healthcare professionals construct agency when discussing patient and family engagement in SI investigations and how these discursive constructions relate to organisational context.

## 2 Materials and Methods

This study is part of a wider research programme titled *The Response Study* (https://responsestudy.leeds.ac.uk/). This study aimed to evaluate, in real time, the implementation of the PSIRF. As part of this evaluation, Phase 2 explored how patient safety incidents were responded to, investigated and learned from within the English NHS under the SIF [13]. Within this phase, a secondary analysis was conducted focusing on professionals' accounts of patient and family engagement.

### 2.1 Data Collection

Phase 2 of the Response Study involved one‐off semi‐structured interviews with NHS staff from six NHS Trusts between July 2023 and April 2024. Trusts participating in the study include three mental health Trusts and three acute Trusts from three NHS England and Improvement (NHSEI) regions, reflecting variation in organisation size, diversity of local population and staff demographic information.

Participants were recruited from different professional groups to gain a holistic understanding of approaches and practices for managing patient safety incidents, including clinical, patient safety, senior management and governance teams. We interviewed between 5 and 10 professionals per Trust, totalling 49 participants (Table 1). Interviews were conducted via Microsoft Teams by Authors 1 and 3, each lasting between 25 and 40 min.

**Table 1:** Phase 2 sample size.

	Trust 1	Trust 2	Trust 3	Trust 4	Trust 5	Trust 6	Total
Patient safety teams	2	4	1	4	3	1	15
Clinical teams	2	4	6	—	3	1	16
Senior leadership, management and governance teams	5	2	2	2	4	3	18
Total	9	10	9	6	10	5	**49**

*Note:* Total sample shown in bold.

The topic guide focused on understanding the management and processing of patient safety incidents under the SIF, exploring the ‘who’, ‘what’ and ‘how’ of investigations. With respect to patient and family engagement, we specifically asked how SIF processes impacted patients, families and service users; how effectively they addressed fairness and equality; how those affected by a safety incident were involved in the investigation process; and whether they were notified or involved in any subsequent changes/improvements following the investigation.

The study received ethical approval from the London‐Stanmore Research Ethics Committee (22/PR/1600). All participants provided written consent prior to the interviews. The exact professional roles are omitted here to preserve anonymity.

### 2.2 Discourse Analytic Approach

We adopted a discourse analytic approach informed by Interactional Sociolinguistics (IS) to examine how healthcare professionals constructed agency when discussing patient and family engagement in SI investigations. IS views meaning as socially situated and focuses on how speakers use linguistic resources to construct social identities and relationships within specific institutional contexts [14]. Previous healthcare research has employed discourse analysis with interview data to examine how participants construct and negotiate meaning, for example, in studies of healthcare professionals' views on digitally supported person‐centred care [15] and patient discourses on real‐time access to test results [16]. While our analysis considered how organisational context shaped participants' accounts, the focus was on identifying recurring linguistic patterns through which participants constructed agency, responsibility and professional identity.

Consistent with IS approaches, our analysis involved examining interview data in fine‐grained ways, focusing not only on what was said but also on how meanings were constructed through language use. This included a close reading of word choice and phrasing, sentence structure and grammar, repetitions, emphatic speech, and how speech sequentially unfolds—that is, what comes after what [17, 18]. Through this micro‐level analysis, we were able to identify *patterns*—structures/linguistic features that consistently appeared across the interviews and are presented in Section Results, 3. By moving beyond what people say to how they say it, our approach revealed how staff's language constructs powerful and powerless professional identities in the context of patient safety and how these identities influence their actions. The concept of agency, discussed below, provided a sensitising lens through which these discursive constructions were interpreted.

### 2.3 Agency as a Sensitising Concept

This study draws on the concept of agency as a sensitising concept. Individual agency, a core but nebulous concept in social sciences, is shaped and constrained by structural factors [19], providing a valuable lens for analysing how healthcare professionals navigate the complex organisational dynamics that shape patient and family engagement. Instead of viewing engagement as the result of institutional policies or individual preferences, an agency‐based approach recognises how social actors actively interpret, negotiate and potentially transform their organisational realities through discursive practices [20]. This perspective is particularly relevant in the NHS, where professional roles, organisational hierarchies and patient safety policies intersect. By examining how NHS staff linguistically construct agency—positioning themselves as either empowered or bound by external constraints—we can better understand the persistent challenges in meaningful patient and family involvement.

## 3 Findings: Contrasting Discourses in Patient and Family Engagement

The data revealed two prevailing but contested discourses: one depicting Trusts' engagement with patients and families in SI investigations as inconsistent and limited, and the other emphasising their integral role in the investigation process. Interviewees consistently framed themselves as either agentless (powerless) or agentive (powerful), aligning with these respective discourses, while organisational factors also fed into those constructions. Participants consistently adhered to one of the two discourses; we found no instances in the interview data of individuals shifting between or combining the two discourses (e.g., related to different investigations). We unpack these discourses along with the linguistic strategies employed to construct them below.

### 3.1 Inconsistent Engagement With Patients and Families Due to External Pressures

The dominant discourse in the data was suggestive of the limited and inconsistent way in which Trusts engage with patients and families. Interviewees reported inconsistencies regarding both the extent of patients/families' involvement in the investigation itself, as well as the information provided to them following its completion, including any decisions on improvement and practice change. Participants reflected on how patients and families would be notified about the investigation, but they would then play a peripheral role in the process, often limited to receiving the final report and/or having the opportunity to raise questions at that final stage. Not all patients and families were given the opportunity to speak to the investigators and share their experiences, which was commented as a significant omission. This inconsistent approach to patient and family engagement was repeatedly attributed to external, impersonal forces—primarily centrally mandated metrics. Those metrics encompassed limited investigative resources, time constraints and the pressure to complete investigations. The overall inflexible nature of the policy itself was seen as a key barrier. Importantly, this discourse was most often employed by frontline clinical and patient safety staff, whose accounts reflected a sense of helplessness and tension with senior organisational teams.

By framing these external forces as the driving agent, interviewees distanced themselves from responsibility, portraying themselves as powerless. This discourse was constructed through: (a) deterministic and negative evaluative language, (b) agentless syntactic constructions, (c) emphatic speech and (d) constructions of otherness.

(a) **Deterministic and Negative Emotive Language**
  Lexical choices within this discourse indicated a preference for deterministic language, implying that interviewees' actions were determined by external forces rather than personal choice. Items constructing this discourse of powerlessness included terms foregrounding a lack of agency, such as ‘bound’, ‘restricted’, ‘limited’, ‘constrained’ and ‘forced’. These terms were primarily used to justify the lack of patient/family engagement, often citing the SIF's ‘arbitrary’ timelines, particularly a 60‐day timeframe for investigations, as illustrated in Quotes 1–2.
  Quote 1
  But I also think some of that's time because **we've been bound by 60 days** in the framework and sometimes that just isn't enough time to get underneath maybe.
  Patient safety team member, Trust 1
  Quote 2
  On occasion under SIF, because **we're constrained by the arbitrary 60‐day rule**, **we don't get the chance** to share a draft report with the family.
  Patient safety team member, Trust 4
  Systematic use of negative evaluative language, broadly understood here as the range of linguistic strategies used to express language users' attitudes or stances, was also prominent. Interviewees described the process of involving patients and families in the investigation as ‘challenging’, ‘overwhelming’ and ‘difficult’ due to the prescriptive nature of the SIF, as shown in Quote 3.
  Quote 3
  It can be **very overwhelming**. Really what they want (the patients/families) is to get down to the meat, really, and say, ‘Well, what happens? What are you going to do differently?’. They just want that. […] it can be **very prescriptive** in having to fill in all these different sections.
  Clinical team member, Trust 2
(b) **Agentless Syntactic Constructions**
  Another discourse strategy employed by participants to reduce their perceived accountability was the use of agentless syntactic constructions—a strategy well‐established in the literature for obscuring/removing human agency and shifting responsibility away (e.g., [21]). Patient safety teams repeatedly talked about how ‘investigations were handled’, rather than how *they* handled them, and how ‘patients were sometimes overlooked’.
  A common form of agency reduction was *nominalisation*, where actions were presented as abstract processes rather than the actions of identifiable individuals (e.g., ‘decisions were made’ rather than ‘we decided’).
(c) **Constructions of Otherness**
  Participants also established in‐group/out‐group distinctions, distancing themselves from other internal teams and/or external stakeholders (e.g., coroners, Integrated Care Boards [ICBs—these are NHS organisations responsible for planning health services for their local population], and the CQC). This was mostly accomplished using personal pronouns, which created contrasts such as ‘I/us’ vs. ‘them’ (on language as a source of otherness and the construction of in‐groups and out‐groups in the workplace, see [22]). This linguistic separation suggests a perception of external forces as intrusive or misaligned with participants' own professional and organisational realities. Quote 4 is an illustrative example, as the interviewee describes how they are not responsible for any learning:
  Quote 4
  Once we've done that feedback, then **it does fall with the patient safety** (team) to track that learning to gather the evidence to close the action plan down. So then after that initial phase, **we leave it to them**.
  Clinical team member, Trust 2
(d) **Emphatic Speech**

The last strategy we identified was emphatic speech, which reinforced some of the above strategies, including constructions of otherness and agentless syntactic structures. In our corpus, emphatic speech manifested through (a) lexical repetition for reinforcement and (b) the use of intensifiers, highlighting the difficulties interviewees faced when interacting with patients and families due to external forces beyond their control (e.g., ***really** difficult, **so** hard, no choice **at all**; **very** overwhelming—*cf. Quotes 3 and 5). Emphatic speech was used in a similar way in support of powerfulness; we return to this in Section 3.2.

Together, these linguistic strategies construct a discourse of powerlessness in which responsibility for involving patients and families is attributed to external factors. Quote 5 illustrates how these patterns intersect.

Quote 5 [Patient Safety Team member, Trust 5]

**Table:** 

**Quote 5**	**Linguistic strategies**
*And I think **people are bound to that 60‐day timeframe** aren't they? So, they feel that if they've submitted a report and then the family come maybe 2 or 3 weeks later and say ‘actually we really want to be part of that investigation process now’*	Responsibility shift towards externally imposed metrics; lexical choice (*bound*) denoting lack of agency
***our ICB sometimes*** weren't ***really helpful in terms of supporting us** with reopening or actually saying we want to keep this open because we want to review in line with the family. […]*	*They* vs. *us*—external stakeholder (ICB) vs. patient safety team
*it was felt that **you were owing to a process** of having to close your incident*.	Inanimate process in an agentive role—participant portrayed as powerless
***So it's difficult. It's difficult really.***	Emphatic speech: lexical repetition of negative evaluative language (*difficult*) + intensifier (*really*)

In the next section, we turn to the second discourse in our data.

### 3.2 Consistent Engagement With Patients and Families Due to Individuals

The second, less frequently employed, discourse in our corpus was the consistent involvement of patients and families in SIs. Here, interviewees emphasised meaningful involvement throughout the investigation process, from the decision to conduct an SI and the preparation of the investigation report to updates on improvement actions.

In contrast to what was the case in the powerlessness discourse, in this case, patient and family involvement was attributed to local factors, with interviewees foregrounding the role of capable staff, strong work relationships and professional experience. This discourse was most often employed by senior leadership and management staff, reflecting their greater authority to shape practice. This aligns with the findings in Section 3.1 and highlights how individuals higher in the professional hierarchy generally feel more in control and possess the authority to shape practice—and are thus better positioned to construct discourses of authority and control.

This discourse was constructed through (a) positive evaluative language, (b) active voice, (c) emphatic speech and (d) constructions of otherness.

**(a) Positive Evaluative Language**

In reflecting on their role in engaging with patients and families, interviewees employed positive evaluative language to express their attitudes to their teams and colleagues, including self‐praise. They described their teams and individual approach in safety investigations as ‘excellent’ and ‘spot on’, noting that it ‘worked really well’. In Quote 6, reflecting on the Trust's involvement of patients and families, a member of the leadership team makes an explicit distinction between the framework (SIF) and the individuals applying it, while in Quote 7, another leadership team member praises their approach as ‘spot on’, using a range of emphatic strategies, which we further unpack later.

Quote 6

When you got someone investigating your incident who **really understood what it was to be open and transparent with the family, really put them, their voice in the centre of the report.** And I have seen it done where **it's done really well** and it's actually the engagement…. I've seen pockets where **it's been absolutely excellent.** But I think **that's more owing to the individuals carrying out the review … than the framework itself.**

Leadership Team member, Trust 1

Quote 7

We will discuss that with the family, let them know that's what we're thinking […] and we'll be really transparent and open about it. And you've got to have that **bravery and your ability to do that**. […] But yeah, **our approach to it has been absolutely spot on and we've used it in the right way and we've made the best, I think, of the framework.**

Leadership Team member, Trust 2

**(b) Use of Active Voice**

Unlike the powerlessness discourse, participants foregrounded themselves as active agents, using active voice to emphasise responsibility, competence and impact (e.g., ‘I led the family liaison’ and ‘I was responsible for keeping the family happy’).

Quote 8 begins with a patient‐focused construction (‘they're fully involved’) before shifting to active voice that foregrounds the professional's agency (‘we share’ and ‘we're doing’):

Quote 8

They're fully involved [the patients] in lots of ways, really, not only through the early learning stuff. **We share** very clearly what **we're doing** and what **we're learning** from it, and **we're very open and honest** about it.

Leadership Team member, Trust 3

**(c) Emphatic Speech**

In Section 3.1, it became apparent how emphatic speech was employed to amplify the difficulties of involving patients/families in SIs. Here, emphatic strategies were also employed, this time, to foreground interviewees' individual and team contributions to successful patient and family involvement. Emphatic strategies identified here include the two already mentioned above: (a) lexical repetition for reinforcement and (b) use of intensifiers: ***really** well* and ***absolutely** excellent*, Quote 6; ***absolutely** spot on*, Quote 7. Importantly, the emphatic use of pronouns (*I **myself***; *the investigators **themselves***) was also observed, while it was absent in the powerlessness discourse. This use of emphatic pronouns serves as another linguistic strategy that foregrounds the subject and highlights their individual contributions to a positive outcome—in this case, meaningful engagement. This is exemplified in Quote 9, where a member of the leadership team narrates what their role involved:

Quote 9

**I myself** would ensure they (patients/families) are kept in the loop.

Another example is Quote 11, where a senior member of the patient safety team narrates how the degree of involvement in investigations depended on ‘the actual investigator **themselves**’.

**(d) Constructions of Otherness**

As in the previous discourse, participants drew distinctions between themselves and others; this time, however, linguistic separation served as a mechanism to foreground the positive impact of individual experiences and work relationships, as opposed to colleagues with less experience, fewer connections and so forth.

Quotes 10 and 11 illustrate how these linguistic strategies intersect to foreground individual agency.

Quote 10 [Leadership team member, Trust 1]

**Table:** 

**Quote 10**	**Linguistic strategies**
*And I think a lot of it is **the confidence of the practitioners** that are undertaking the reviews […] **it's your experience with families** as well. […] if I had one incident where I knew particularly that there was a lot of failings in care, that the family were going to be very aggrieved in terms of the process, and it was gonna be a really awful coroner's experience for anybody involved*	Emphasis on individuals Emphasis on individual work experience
***I'd put my best investigator on that case***	Foregrounding individual contribution
*who I knew could **really** engage with families*.	Intensifier (*really*)

Quote 11 [Patient Safety team member, Trust 5]

**Table:** 

**Quote 11**	**Linguistic strategies**
*And it would also be based **on the actual investigator themselves** (the level of patient involvement)*.	Emphasis on individuals: intensifier (*actual*) + emphatic pronoun (*themselves*)
*And **I had been doing investigations for a long time and I was quite comfortable** to sort of go and ask people and staff and relatives and pay them a visit to ask this. So the recall of what had happened, whereas **not sure that everyone used to do that**, if I'm being honest […]And that*	Emphasis on work experience I *vs.* them I *vs.* them
*was usually **my best approach**, really. I felt **that was the better way**. I preferred to do that, I have to say,*	Self‐praise Self‐praise
***but that was an individual thing***.	Emphasis on individual contribution

Before moving to Section Discussion, 4, Table 2 provides a summary of the contrasting discourses identified in the data, highlighting the linguistic strategies used to construct them and the organisational context that both shapes and is shaped by them.

**Table 2:** Key discourses, supporting linguistic strategies and organisational context.

Dimension	‘Disengaged’ discourse	‘Engaged’ discourse
Patient/Family Involvement	Inconsistent involvement of patients and families	Consistent involvement of patients and families
Agency & Responsibility	Participants portrayed as agentless; responsibility shifted to abstract organisational concepts (processes and timelines) and external factors	Participants in an agentive role, responsible for patient/family engagement
LINGUISTIC STRATEGIES		
*Lexical Choices*	Deterministic language indicating lack of agency—*bound, restricted, limited, forced* Negative evaluative language—*challenging, overwhelming process*	Positive evaluative language and self‐praise—*excellent, spot on*
*Syntax*	Agentless syntactic constructions, including passive voice and nominalisation—*investigations are conducted; the implementation of the policy*	Syntactic constructions foregrounding the subject, including active voice—*I led the family liaison*
*Emphatic Speech*	Lexical repetition Intensifiers—*absolutely*, *completely* Emphatic use of pronouns—*the investigators themselves*	
*Constructions of Otherness*	In‐group/out‐group separation—*I/us vs. them*	
ORGANISATIONAL CONTEXT		
*Role Type*	Primarily employed by staff in hands‐on roles in investigations (e.g., clinical investigation teams)	Primarily employed by staff in senior management/leadership roles
*Work Experience*		Long work experience within a specific team positioned staff as agentive
*Trust Type*	No consistent differences between Acute and Mental Health Trusts or different‐sized Trusts	

## 4 Discussion

In examining how staff discursively construct agency in their accounts of engaging patients and families in SI investigations, our findings revealed two contrasting discourses. The first one is their inconsistent and limited engagement, which was largely attributed to external forces. This dominant discourse aligns with previous research on barriers faced by healthcare professionals [5] and absent policy support [7]. The second, less common discourse emphasised the consistent and meaningful involvement of patients and families, attributing it to local factors and professionals' agency. This depiction of patients and families as integral to the entire investigation process was not as visible in previous thematic analyses, highlighting how micro‐analytic approaches such as the one taken here can help us identify and interpret nuanced patterns of meaning.

The two discourses were underpinned by distinct linguistic patterns that positioned interviewees as either agentless or agentive. Limited involvement was associated with deterministic language, agentless constructions and othering processes, whereas meaningful involvement was linked to positive evaluation, active voice and explicit claims to responsibility. The examination of the organisational context illustrated that professional roles both shaped staff's accounts and were reflected in them: frontline staff from clinical and patient safety teams largely aligned with the discourse of powerlessness, while senior staff from leadership and management teams emphasised a discourse of authority and control. This reflects the NHS's hierarchical leadership structure, which has been shown to disconnect managers from frontline staff and risk patient safety [23].

Our findings have significant implications for the future involvement of those affected by safety incidents. The ways in which staff linguistically construct agency, power and responsibility can influence engagement efforts, shaping both constraints and opportunities for meaningful involvement. Our micro‐analysis highlights the problems and possibilities created by different discourses, as discussed by Hulter et al. [16]. Our analysis showed that staff directly involved in investigations tend to perceive their role as relatively passive, feeling constrained by external organisational factors. These findings align with Sheard et al.'s [24] large qualitative study showing weak ‘structural legitimacy’ amongst frontline NHS staff, limiting their ability to effect change. This discursive construction of ‘helplessness’, in turn, feeds into their everyday practice, further hindering meaningful engagement. From a practical perspective, these findings suggest that efforts to improve patient and family involvement should extend beyond procedural changes and address how staff understand their own role in the engagement process. Reflective training, supervision and investigation debriefs could help staff identify assumptions about responsibility and control, while creating opportunities for frontline staff to contribute to decisions about investigation processes, which may strengthen their sense of agency and capacity to engage patients and families meaningfully.

Conversely, those in senior management and leadership roles position themselves as highly agentive, feeling in control of the situation—yet this sense of control may paradoxically limit meaningful improvement, as their experience of involvement differs significantly from that of frontline staff. Our study thus provides further evidence of this separation through linguistic analysis, highlighting the role that discourse analytic approaches can play in triangulating more traditional qualitative methods.

Importantly, the disconnect between these discourses and the groups that employ them points to deeper structural issues, including hierarchical barriers and team fragmentation. Such issues are pervasive in healthcare organisations [25]. Identifying distinct discourse patterns, as we did here, can potentially uncover barriers to meaningful involvement, inform training that encourages more agentive participation, and support policies aimed at fostering greater transparency and collaboration in patient safety investigations.

Turning to limitations, a key methodological critique of interviews is that they often have a performative dimension, with interviewees shaping their responses based on what they believe the interviewer expects to hear (on performative talk, see [26]). In a hierarchical system like the NHS, these performative aspects may be further amplified when discussing critical topics such as patient and family involvement. Nevertheless, such insights are valuable in themselves, as they reflect staff perceptions of role expectations and performance (e.g., what a Director of Patient Safety *should* be doing, rather than what they do). Secondly, patients and families were not included as participants in this study. Our research question specifically examined healthcare professionals' constructions of their own roles and agency in patient and family engagement. Including patient perspectives would have shifted the focus to experiences of engagement rather than professional identity construction. Nevertheless, a Citizens' Panel was involved throughout the Response Study, supporting wider discussions about the emergent findings of the research.

## 5 Conclusion

While qualitative health research has largely prioritised content and thematic analyses, linguistic approaches remain underutilised, especially in interview studies, as they are typically reserved for ‘real‐life’ interactions. Our study demonstrated how discourse analytic approaches can yield important insights into the social and organisational realities that shape patient and family involvement in SI investigations. An analytical focus on the discursive construction of agency sheds light not only on individual professional experiences but also on the broader institutional structures that enable or limit transformative practice in healthcare organisations. By uncovering two distinct discourses surrounding patient and family engagement—linked to professional roles, perceived agency and broader institutional constraints—we demonstrated how language reflects and reproduces existing hierarchies and expectations within the NHS. These discourses do not merely describe professional practice; they help constitute it, shaping what staff see as doable or permissible within their roles. Focusing on the linguistic construction of agency provides a powerful lens for understanding persistent gaps in patient and family engagement and highlights the value of incorporating discursive approaches into health policy development and implementation. Future research should explore how discourses of agency are reproduced, challenged and transformed within healthcare organisations and how such changes may contribute to more meaningful patient and family involvement in safety investigations.

## Author Contributions

**Polina Mesinioti:** conceptualisation, investigation, writing – original draft, methodology, writing – review and editing, data curation. **Laura Sheard:** writing – review and editing, conceptualisation, methodology, writing – original draft. **Sarah Hampton:** investigation. **Gemma Louch:** project administration, writing – review and editing. **Carl Macrae:** funding acquisition, writing – review and editing, conceptualisation. **Jane O'Hara:** funding acquisition, writing – review and editing, conceptualisation.

## Ethics Statement

The study received ethical approval from the London‐Stanmore Research Ethics Committee (22/PR/1600). Participants were informed that their data may be used in publications and presentations.

## Consent

All participants provided written consent prior to the interviews.

## Conflicts of Interest

The authors declare no conflicts of interest.

## Data Availability

The data that support the findings of this study are available on request from the corresponding author. The data are not publicly available due to privacy or ethical restrictions.
